# Epidemiological Study Based on China Osteonecrosis of the Femoral Head Database

**DOI:** 10.1111/os.12857

**Published:** 2020-12-21

**Authors:** Biao Tan, Wenlong Li, Ping Zeng, Haoshan Guo, Zeqing Huang, Fanyu Fu, Huanhuan Gao, Rongtian Wang, Weiheng Chen

**Affiliations:** ^1^ Department of Orthopaedics Wangjing Hospital, China Academy of Chinese Medical Sciences Beijing China; ^2^ The Third Affiliated Hospital of Beijing University of Chinese Medicine Beijing China; ^3^ Second Department of Orthopaedics Xianhu Branch of the First Affiliated Hospital of Guangxi University of Chinese Medicine, Guangxi Zhuang Autonomous Region Nanning China; ^4^ Department of Orthopaedics Liaocheng Traditional Chinese Medicine Hospital of Shandong Province Liaocheng China; ^5^ Guizhou University of Traditional Chinese Medicine Guiyang China

**Keywords:** Case registration system, Epidemiology, Etiology, Osteonecrosis of the femoral head

## Abstract

**Objective:**

The aim of the present study was to reveal the case characteristics of osteonecrosis of the femoral head (ONFH) in Mainland China.

**Methods:**

This cross‐sectional epidemiological study derived data for ONFH patients from July 2016 to December 2018 from the China Osteonecrosis of the Femoral Head Database (CONFHD). The derived data included gender, age, body mass index (BMI), height, occupation, region, and etiology of femoral head necrosis. A descriptive analysis was performed to summarize the epidemiological characteristics of the case data in the CONFHD.

**Results:**

A total of 1844 ONFH patients (2945 hips) were included in this study, comprising 1302 men and 542 women. The age of patients ranged from 18 to 95 years, with a median of 50 years, and the male to female ratio was 2.4. Male patients are younger than female patients (48.26 ± 12.56 years old and 55.56 ± 14.94 years old, respectively). Among the 1844 patients (2945 hips), there were 528 (17.92%) hips at ARCO stage I, 941 (31.99%) hips at ARCO stage II, 873 (29.63%) hips at ARCO stage III, and 603 (20.46%) hips at ARCO stage IV. In the subclassification of ARCO stages I and II, the majority of cases were type C; type A comprised the majority in the subclassification of ARCO stage III. According to the Kellgren–Lawrence classification system, among the 603 ARCO stage IV hips, there were 178 (29.52%) grade 1 hips, 201 (33.34%) grade 2 hips, 176 (29.18%) grade 3 hips, and 48 (7.96%) grade 4 hips. Most were from three provinces: Henan (27.3%), Shanxi (13.9%), and Shandong (11.9%). Regarding BMI, 982 patients (53.25%) were overweight or obese. Among all patients, the largest proportion of patients engaged in level IV manual work. Of all the patients, there were 495 (26.84%) with steroid‐induced ONFH, 685 (37.15%) were alcoholics, and 290 (15.73%) had traumatic ONFH. The 495 patients with steroid‐induced ONFH included 278 men (56.16%) and 217 women (43.84 %), had a complete history of hormone use. Among the primary diseases, there were 195 cases (39.39%) of immune system diseases, followed by dermatological diseases, respiratory diseases, nephropathy, and other diseases. There were a total of 685 patients with alcoholic ONFH, 589 of these patients (85.99%) were men. A total of 188 (27.45%) patients had drunk alcohol for 6–10 years (comprising the highest proportion), and 280 patients (40.88%) consumed 3001–3500 mL of alcohol each week (the highest proportion).

**Conclusion:**

Osteonecrosis of the femoral head most commonly occurs after the age of 40. Male patients have an earlier onset than female patients, and the number of male patients is approximately twice that of female patients. The BMI of patients was mainly in the overweight and obese range, and half of these patients engaged in level IV manual work. From the imaging findings, the numbers of hips at ARCO stages II and III were greatest, and the number at stage I was relatively small. Among all the causes of disease, alcohol, steroid use and trauma were the three most common reasons for ONFH.

## Graphical abstract

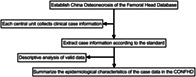

This is the first Internet‐based ONFH patient case registration study for mainland China. It presents epidemiological characteristics of osteonecrosis of the femoral head in mainland China.

## Introduction

As a daunting and intractable orthopaedic disease, osteonecrosis of the femoral head (ONFH) can gradually lead to lifelong disability in patients. ONFH primarily affects young to middle‐aged adults^1^. According to Mont *et al*., the total number of people with ONFH worldwide will reach 20 million by next ten years^2^. The annual prevalence in the United States is between 15,000 and 20,000^3^, ^4^, and it is estimated that there are approximately 8.12 million people over the age of 15 with ONFH in China. The extremely high prevalence of ONFH leads to loss of employment and is an economic burden on patients, their families, and society^5^. Therefore, recording various clinical data on patients with ONFH in the form of a case registration system is critical. This would assist researchers in understanding the occurrence, development, outcome, and prognosis of this disease.

A good case registration system can help medical workers and local governments to understand the occurrence and development of diseases, which is conducive to the development of effective strategies for prevention and treatment. Kang *et al*. used medical claims data from the Korean National Health Insurance Corporation to estimate the prevalence of ONFH in Korea^6^. Ikeuchi *et al*. reported^7^ that the annual incidence rate in Japan (with a population of 128 million) was 1.91/100,000 in 2015. Fukushima *et al*.^8^ reported that the peak age of non‐traumatic ONFH in Japan is 40 years, and the etiology is, respectively, steroid use (51%), alcohol consumption (31%), and idiopathic (15%). A survey by Cui *et al*. of nine tertiary hospitals in mainland China showed that the incidence of ONFH is concentrated in individuals between the ages of 40 and 50 years and that men have an earlier incidence than women. Autoimmune diseases are the main cause of steroid‐induced ONFH^9^. Although their study included 6395 cases of ONFH, all of them were inpatients in major tertiary hospitals in mainland China. We believe that this does not fully represent the epidemiological status of ONFH in China. Because in China large hospitals generally treat patients with worse conditions, many patients with mild disease will not be admitted to these hospitals, and a considerable number of patients with ONFH choose to follow up in the outpatient.

In this study, we analyzed the medical records of registered ONFH patients through a paperless network case registration system established previously. This system has already operated in 25 hospitals. This is just the beginning. We hope that this case registration system will be continuously improved and that the analysis of the registered case data will play a role in preventing and treating ONFH in China. The purpose of this study was to reveal the distribution of ONFH in different regions of China, the distribution of ONFH for different population characteristics, and the pathogenic factors and stage distribution of ONFH of different etiologies.

## Materials and Methods

### *Study Design*

The China Osteonecrosis of the Femoral Head Database (CONFHD) is an electronic structured case collection system supported by the National Key Technology Research and Development Program of the Ministry of Science and Technology of China. The CONFHD program was established with the purpose of improving medical management of ONFH patients in mainland China. It is a non‐profit database for medical research use only and defines the rights and obligations of clinical cooperation units on the principle of “mutual benefit, equality, voluntary and open sharing.” Between 2016 and 2018, the CONFHD recruited 2400 ONFH patients through 25 public hospitals from 11 administrative areas (provinces or municipalities) across mainland China. The main sampling areas were Beijing Municipality, Shanghai Municipality, Shandong Province, Henan Province, Guangxi Province, Shaanxi Province, Guangdong Province, Jiangsu Province, Fujian Province, Jilin Province, and Hubei Province. This study is a retrospective cross‐sectional case study approved by the Ethics Committee of Wangjing Hospital, China Academy of Chinese Medical Sciences. Research secretaries were responsible for data entry. The research secretaries were not involved in the medical management of ONFH patients and underwent strict quality control training. The attending doctors were also not involved in this study. Three orthopaedic surgeons who specialized in hip arthritis and who were not involved in the present study were invited to evaluate the images; each decision was made by consensus among the three surgeons. Therefore, the quality of the data can be ensured.

### *Data Collection*

Since 2015, we have prepared and constructed the CONFHD, which is an online case registration system based on the web (http://onfh.keyanyun.com/). Each participating unit has its independent account to facilitate the registration of patients with ONFH and record their basic information, treatment strategies, and follow‐up data. All patient information is privately protected and can only be used by researchers for disease research. We extracted medical records of valid patients with ONFH registered in the CONFHD from July 2016 to December 2018.

The inclusion criteria are as follows: (i) meet the diagnostic criteria for femoral head necrosis proposed by Mont *et al*. in 1995^10^; (ii) patients must be 18 years or older; and (iii) the medical information registered in the case registration system is complete. The exclusion criteria are as follows: (i) irregular and incomplete data; and (ii) duplicate data.

### *Data Structure and Classification*

This Internet‐based case registration system contains information on the following aspects: patient demographics, general conditions, specialist examinations (including physical examinations, laboratory investigations, and imaging findings), treatment process records and follow‐up information.

In this study, we used the following data: gender, age, body mass index (BMI), classification and stages of the diseases, nationality, labor intensity, geographical division, and types of ONFH. We classified ONFH into four categories: traumatic, steroid‐induced, alcoholic, and idiopathic. According to the recommendations of the Obesity Working Group, International Life Science Association of China^11^, we divided the BMI into four levels: (i) BMI <18.5 is considered underweight; (ii) 18.5 ≤ BMI < 24 is considered normal; (iii) 24 ≤ BMI < 28 is considered overweight, (iv) BMI >28 is considered obese. According to the National Labor Standards of the People's Republic of China^12^, we divided labor intensity into four grades: civilians, retirees, and civil servants were classified as grade I; doctors, teachers, drivers, and engineers were included in grade II; electricians and cleaners were grade III; and peasants and workers were grade IV. Freelancers and the unemployed, for example, are classified into other categories. All patients were divided among seven geographical regions: East China, North China, Central China, South China, Southwest, Northwest, and Northeast.

### *Statistical Analysis*

All data conforming to the normal distribution are expressed as mean ± standard deviation, and data for non‐normal distribution are expressed as median (range, 25th, and 75th percentiles). Comparison of the rates was done using the *χ* ^*2*^‐test. A two‐tailed *P*‐value of <0.05 was considered to be of statistical significance. All data were performed using SPSS 26.0 (International Business Machines, Armonk, New York, USA).

## Results

### *Demographic Characteristics of Osteonecrosis of the Femoral Head Patients in China Osteonecrosis of the Femoral Head Database*

**Fig 1:**
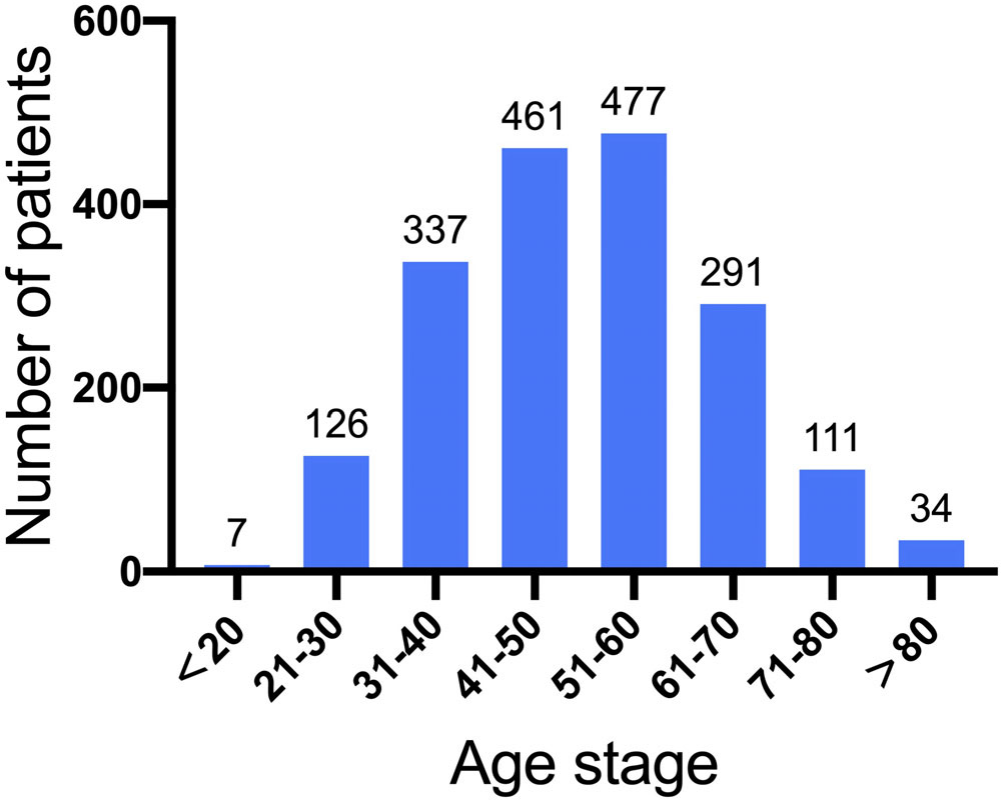
Age distribution of patients with osteonecrosis of the femoral head (ONFH) in China Osteonecrosis of the Femoral Head Database. The age of the patients was mainly between 41 and 60 years. This indicates that ONFH mainly affects young to middle‐aged people.

A total of 1844 patients (2945 hips) were included in the clinical analysis, with complete medical history available. Among them, 1302 (70.6%) were male and 542 (29.4%) were female, with a gender ratio of 2.4:1. The mean age at the time of diagnosis was 50.40 ± 13.71 years (male 48.26 ± 12.56; female 55.56 ± 14.94). The mean age difference was 7.3 (5.97–8.63; *t* = −10.74, *P* = 0.000). The age of patients in the CONFHD is mainly distributed in the two age groups of 41–50 and 51–60 years, accounting for 50.9% of the total number. The age distribution is demonstrated in Fig. 1.

### *Body Mass Index Distribution of Osteonecrosis of the Femoral Head Patients in China Osteonecrosis of the Femoral Head Database*

**Fig 2:**
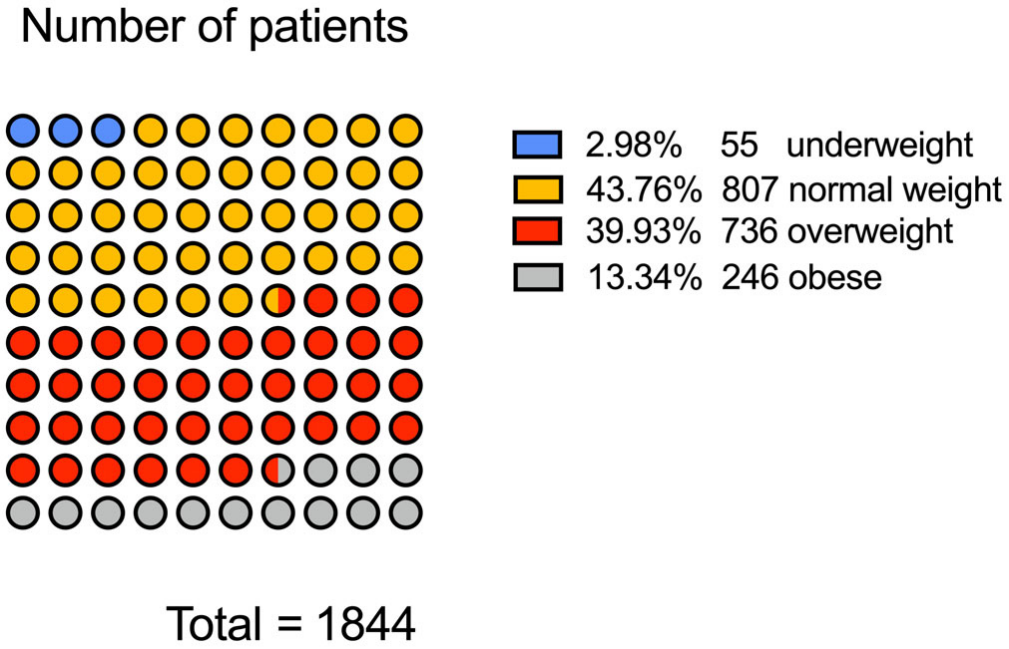
BMI composition of patients with osteonecrosis of the femoral head (ONFH) in the China Osteonecrosis of the Femoral Head Database. More than half of the patients are overweight and obese.

In 1844 patients with ONFH, the mean BMI was 24.36 ± 3.56 (male 24.65 ± 3.42; female 23.68 ± 3.78). The mean BMI difference was 0.97 (0.61–1.32; *t* = 5.35, *P* = 0.000). There were 55 patients who were underweight (2.98%), 807 of normal weight (43.76%), 736 who were overweight (39.93%), and 246 who were obese (13.34%); 982 of patients were overweight or obese (53.25%) (Fig. 2).

### *Disease Stages and Imaging Features of Osteonecrosis of the Femoral Head Patients in China Osteonecrosis of the Femoral Head Database*

According to the classification system of the Association Research Circulation Osseous (ARCO)^13^, MRI was used to assess the hips at ARCO stage I, and X‐rays were used to assess the hips at ARCO stages II, III, and IV. Among the 1844 patients (2945 hips), there were 528 (17.92%) hips at ARCO stage I, 941 (31.99%) hips at ARCO stage II, 873 (29.63%) hips at ARCO stage III, and 603 (20.46%) hips at ARCO stage IV. In the subclassification of ARCO stage I, 98 (18.56%) hips were type A, 156 (29.54%) hips were type B, and 274 (51.90%) hips were type C. In the subclassification of ARCO stage II, 285 (30.29%) hips were type A, 316 (33.58%) hips were type B, and 340 (36.13%) hips were type C. In the subclassification of ARCO stage III, 364 (41.70%) hips were type A, 267 (30.58%) hips were type B, and 242 (27.72%) hips were type C. Detail of ARCO stages and imaging features of ONFH patients in the CONFHD are shown in Table 1. The Kellgren–Lawrence classification^14^ method was used to evaluate the severity of osteoarthritis of 603 ARCO stage IV hips; there were 178 grade 1 hips (29.52%), 201 grade 2 hips (33.34%), 176 grade 3 hips (29.18%), and 48 grade 4 hips (7.96%) (Table 2).

**TABLE 1:** ARCO stages and imaging features of ONFH patients in the CONFHD

Stage	Number of hips	Subclassfication	Subclassfication number
	*N* (%)		*N* (%)
I	528 (17.92%)	A	98 (18.56%)
		B	156 (29.54%)
		C	274 (51.90%)
II	941 (31.99%)	A	285 (30.29%)
		B	316 (33.58%)
		C	340 (36.13%)
III	873 (29.63%)	A	364 (41.70%)
		B	267 (30.58%)
		C	242 (27.72%)
IV	603 (20.46%)	‐	‐
Total	2945 (100.00%)	‐	‐

Note: According to the subclassfication of ARCO stage (area involvement, percentage): A, minimal (<5%); B, moderate (15% to 30%); C, extensive (>30%). ARCO, Association Research Circulation Osseous; CONFHD, China Osteonecrosis of the Femoral Head Database; ONFH, osteonecrosis of the femoral head

**TABLE 2:** Imaging features of ARCO stage IV ONFH patients in CONFHD

Evaluation method	Grade	Number of hips
		*N* (%)
Kellgren–Lawrence classifification	0	0 (0.00%)
	1	178 (29.52%)
	2	201 (33.34%)
	3	176 (29.18%)
	4	48 (7.96%)
In total	‐	603 (100.00%)

Note: Details of Kegelen–Lawrence classification: grade 0, no radiographic features of osteoarthritis are present; grade 1, doubtful joint space narrowing and possible osteophytic lipping; grade 2, definite osteophytes and possible joint space narrowing on anteroposterior weight‐bearing radiograph; grade 3, multiple osteophytes, definite joint space narrowing, sclerosis, possible bony deformity; grade 4, large osteophytes, marked joint space narrowing, severe sclerosis, and definite bony deformity.

ARCO, Association Research Circulation Osseous; CONFHD, China Osteonecrosis of the Femoral Head Database; ONFH, osteonecrosis of the femoral head

### *Regional and Labor Intensity Distribution of Osteonecrosis of the Femoral Head in the China Osteonecrosis of the Femoral Head Database*

**Fig 3:**
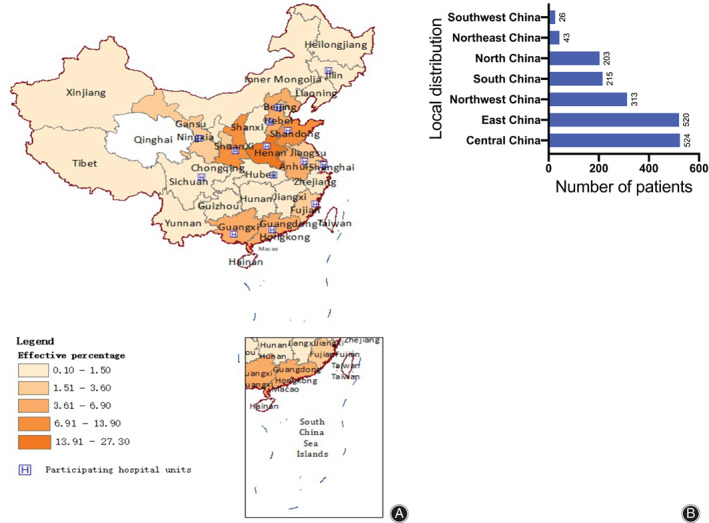
A, Schematic diagram of the regional distribution of patients in CONFHD. The provinces and cities with the largest proportions were: Henan Province, 503 (27.3%); Shanxi Province, 257 (13.9%); Shandong Province, 220 (11.9%); Guangxi Zhuang Autonomous Region, 128 (6.9%); and Hebei Province, 116 (6.3%). B, Bar chart of the regional distribution of patients in CONFHD. The top three regions for the number of patients are: Central China, 524 (28.4%); East China, 520 (28.2%); and Northwest China, 313 (17%).

**Fig 4:**
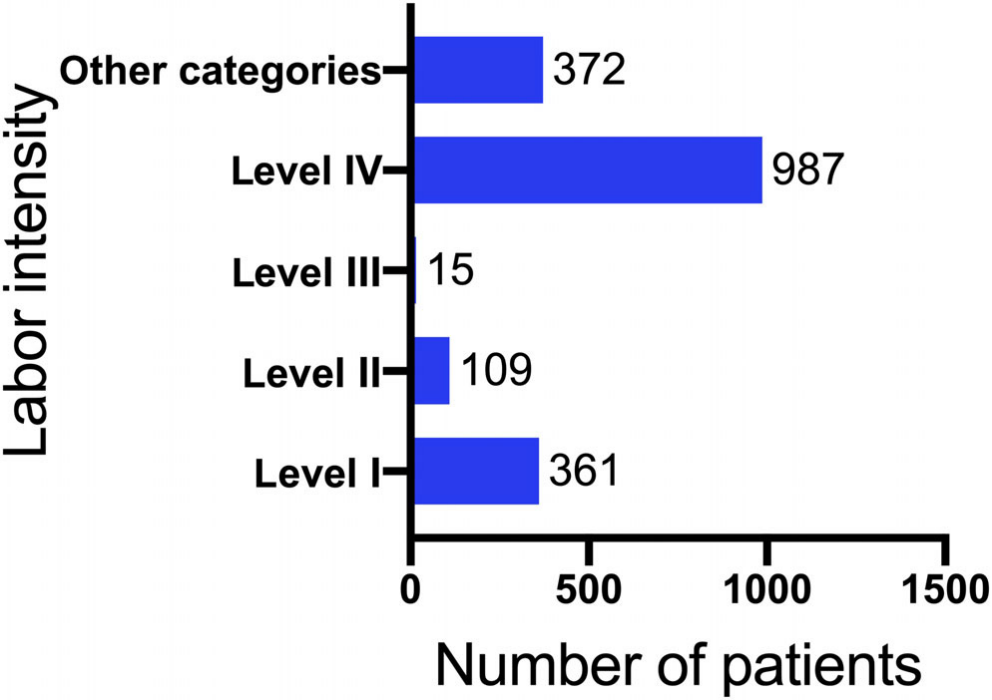
Bar chart of labor intensity distribution of patients engaged in the China Osteonecrosis of the Femoral Head Database. The largest number of patients are engaged in level IV manual work. This implies that heavy physical labor may also be one of the risk factors for osteonecrosis of the femoral head.

Among all 1844 patients, 520 (28.2%) were in East China, 203 (11%) in North China, 524 (28.4%) in Central China, 215 (11.7%) in South China, 26 (1.4%) in Southwest China, 313 (17%) in Northwest China, and 43 (2.3%) in Northeast China. All patients were distributed across 29 provinces, municipalities, and autonomous regions in mainland China. The provinces and cities with the largest proportions were: 503 cases (27.3%) in Henan Province, 257 cases (13.9%) in Shanxi Province, 220 cases (11.9%) in Shandong Province, 128 cases (6.9%) in Guangxi Zhuang Autonomous Region, and 116 cases (6.3%) in Hebei Province. The regional distribution is demonstrated in Fig. 3. In terms of labor intensity, there were 361 cases (19.6%) of level I, 109 cases (5.9%) of level II, 15 cases (0.8%) of level III, and 987 cases (53.5%) of level IV labor intensity; there were 372 cases (20.2%) in other categories (Fig. 4).

### *Etiological Distribution of Osteonecrosis of the Femoral Head in the China Osteonecrosis of the Femoral Head Database*

**Fig 5:**
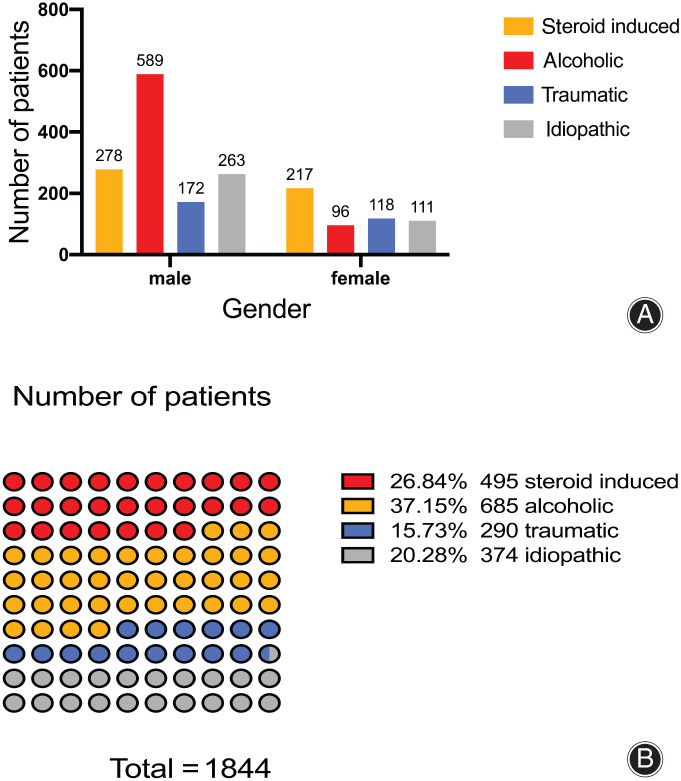
A, Etiological distribution of patients by gender in the China Osteonecrosis of the Femoral Head Database (CONFHD). (Osteonecrosis of the femoral head [ONFH] occurs mainly in men, and the main cause of the disease is alcoholism in men and hormones in women). B, Etiological composition of patients in CONFHD. (The results show that alcoholism and steroid‐induced ONFH are still the main causes of femoral head necrosis in mainland China)

Among all patients, there were 495 (26.84%) steroid‐induced ONFH patients, 685 (37.15%) alcoholic ONFH patients, 290 (15.73%) traumatic ONFH patients, and 374 (20.28%) idiopathic ONFH patients. Gender was associated with different etiologies. In male patients, alcoholism was the most common etiology (45.2%), while in female patients, steroid‐induced and traumatic were the major etiologies (40% and 21.8%, respectively) (Fig. 5).

There were 495 patients with steroid‐induced ONFH with a complete history of hormone use, including 278 (56.16%) men and 217 (43.84%) women. The primary diseases included: 195 cases (39.39%) of autoimmune disease, 71 cases (14.34%) of dermatological disease, 58 cases (11.72%) of respiratory disease, 53 cases (10.71%) of nephrotic disease/nephritis, 47 cases (9.50%) of hematological disease, 27 cases (5.45%) of bone and muscular diseases, 24 cases (4.85%) of neurological disease, 10 cases (2.02%) of ear, nose and throat system diseases, and 10 cases (2.02%) of other diseases. The underlying illnesses of steroid‐induced ONFH are shown in Table 3.

**TABLE 3:** Underlying illnesses of steroid‐induced ONFH

Primary diseases	Number of occurrences	Ratio (%)
Autoimmune disease	195	39.39
Systemic lupus erythematosus	156	31.52
Henoch–Schonlein purpura	13	2.62
Rheumatoid arthritis	11	2.22
Multiple sclerosis	8	1.62
Ankylosing spondylitis	7	1.41
Dermatological disease	71	14.34
Respiratory disease	58	11.72
Nephrotic disease/nephritis	53	10.71
Hematological disease	47	9.50
Bone and muscular diseases	27	5.45
Neurological disease	24	4.85
Ear, nose, and throat disorders	10	2.02
Other	10	2.02
In total	495	100.00

ONFH, osteonecrosis of the femoral head

There were 685 patients with alcoholic ONFH with complete drinking history, including 589 (85.99%) men and 96 (14.01%) women. Among them, 45 patients (6.57%) starting drink within the previous 5 years, 188 (27.45%) had been drinking for 6–10 years, 52 (7.59%) had been drinking for 11–15 years, 186 (27.15%) had been drinking for 16–20 years, 24 (3.50%) had been drinking for 21–25 years, 94 (13.72%) had been drinking for 26–30 years, 5 (0.73%) had been drinking for 31–35 years, 14 (2.05%) had been drinking for 36–40 years, and 77 (11.24%) had been drinking for more than 40 years. Table 4 presents the years of drinking and the amount of drinking per week.

**TABLE 4:** Patients with alcoholic ONFH drinking years and weekly drinking amount

Terms	Number of cases	Ratio (%)
Drinking years		
≤5	45	6.57
6–10	188	27.45
11–15	52	7.59
16–20	186	27.15
21–25	24	3.50
26–30	94	13.72
31–35	5	0.73
36–40	14	2.05
>40	77	11.24
In total	685	100.00
Weekly alcohol consumption (mL)		
≤500	6	0.88
501–1000	35	5.10
1001–1500	14	2.04
1501–2000	32	4.67
2001–2500	38	5.55
2501–3000	97	14.16
3001–3500	280	40.88
>3500	183	26.72
In total	685	100.00

ONFH, osteonecrosis of the femoral head

## Discussion

Osteonecrosis of the femoral head is an orthopaedic disease with a very high disability rate. Both joint replacement therapy, represented by total hip arthroplasty, and hip‐preserving treatment, represented by core decompression, have made great progress^15^, ^16^. In mainland China, orthopaedic surgeons can also choose Chinese medicine as a treatment method for patients^17^, ^18^. Considerable experimental work has been done in this area^19^, ^20^, ^21^, ^22^, and a large number of patients who received hip‐sparing therapy with integrated medicine achieved satisfactory results^23^. However, the epidemiology of ONFH has not been as thoroughly investigated as its treatment; studying its epidemiology is particulary important for a country like China with a large land area and population. To our knowledge, this is the first Internet‐based case registration study of patients with ONFH in mainland China. Our main work from 2015 was to build this web‐based database, and we have included the information of 1884 patients from the first 25 hospitals that joined the database.

Of the 1844 patients included in this study, 70.6% were men, 29.4% were women; the male to female ratio was 2.4:1. This is consistent with the results of a 2009 study of the incidence of ONFH in Korea by Kang *et al*.^6^. Such results are close to the results of a survey of nine large tertiary hospitals in China conducted by Cui *et al*.^9^. These results indicate that men have greater incidence of ONFH than women, and men are younger than women at the time of illness.

Obesity has been proven to be a risk factor for many diseases^24^, ^25^, ^26^. Although body mass has a positive effect on bone formation, whether the mass derived from obesity or excessive fat accumulation is beneficial to bone remains controversial. Obesity possibly affects bone metabolism through several mechanisms^27^. In this study, 53.2% of patients were overweight or obese. Based on such results, we speculate that an excessively high BMI may adversely affect the occurrence of ONFH, but such conjecture needs to be confirmed with further research.

From the imaging findings, the numbers of hips at ARCO stages II and III were the greatest; the numbers at stage I were relatively small, which might indicate that in the early stage of the disease, when the patient has not yet shown clinical symptoms, it is not easy to diagnose by plain radiographs, and there is even the possibility of misdiagnosis. In the subclassification of ARCO stages I and II, the majority were type C. However, in the subclassification of ARCO stage III, type A formed the majority. This may indicate that early disease detection and intervention can delay the natural development of the disease.

In this study, 53.5% of workers were engaged in level IV labor. People with higher labor intensity have a greater hip joint load, which leads to a higher collapse rate; the diet and lifestyle of these individuals also make their incidence rates higher than those engaged in other occupations. In addition, due to the generally low level of education and poor economic status of level IV manual laborers, they lack awareness of ONFH and are likely to miss early treatment opportunities. This conclusion coincides with Japanese scholars’ view that heavy physical work will adversely affect the occurrence of femoral head necrosis^28^.

Our research shows that steroid‐induced ONFH and alcoholic ONFH are the main types of femoral head necrosis. However, patients with alcoholic femoral head necrosis still dominate. We believe that this is because most of the registered patients are from the major drinking provinces in Central, Eastern, and Northwest China.

The results of our study showed that alcohol accounted for the highest proportion of male ONFH patients, followed by hormonal and traumatic ONFH, suggesting that alcohol has become the most important cause of male ONFH. Some studies have shown that ethanol can not only inhibit osteoblast proliferation and differentiation, accelerate osteoblast apoptosis, but also induce precursors to adipogenic differentiation, resulting in bone homeostasis imbalance. At the same time, ethanol will cause blood lipid metabolism disorder, promote fat production, and increase intraosseous pressure, resulting in obstruction of blood supply in the femoral head^29^. In this study, approximately 95% of the patients had drunk alcohol for more than 5 years, more than half of the patients drank more than five times a week, and most patients drank more than 3000 mL per week. Most patients with alcoholic ONFH have the characteristics of long‐term, heavy, and frequent drinking. Therefore, it is necessary to increase publicity and education regarding the relationship between alcohol and ONFH, to reduce alcohol intake and actively prevent and ONFH.

Long‐term or extensive use of hormones has become the main risk factor for inducing non‐traumatic ONFH^30^. The number of ONFH cases induced by hormones is increasing year by year, which is mainly related to the increase in the number of patients with a primary disease treated with hormones, and the inappropriate use of hormones. In this study, there are many primary diseases related to steroid‐induced ONFH, including autoimmune, dermatological, respiratory, nephrotic, and hematological diseases. Excessive use of hormones is an important cause of ONFH. The primary diseases, such as low back pain, gouty arthritis, otitis media, upper respiratory tract infection, and fever, that do not require long‐term and extensive use of hormones, suggest the serious consequences of excessive use of hormones. If the hormones are used appropriately, the occurrence of ONFH can be avoided to a certain extent.

Our study has several limitations. First, although 25 hospitals have been included so far, there are not many registered patients, and the distribution of these hospitals in mainland China is uneven, resulting in a certain bias in the geographical distribution of patients. Second, although the hospitals participating in this database are all tertiary hospitals, they are mainly Chinese medicine hospitals, so there will be some bias in the choice of hospitals. However, as mentioned earlier, establishing CONFHD is a good start for patients in mainland China with femoral head necrosis and for the government. In future work, we will gradually overcome these difficulties and shortcomings, Improve CONFHD, do some practical work on the epidemiological causes of femoral head necrosis in patients in China, and promote our understanding of the disease.

### *Conclusion*

The most common age range when ONFH occurs is 41 to 60 years. Male patients developed ONFH 7.3 years earlier than female patients. The sex ratio between men and women is approximately 2.4:1. The proportion of overweight and obese patients and level IV manual laborers is relatively high, and the patients are mainly concentrated in central, eastern, and northwestern China. Of all causes, alcohol, steroid use, and trauma are the three most common.
